# Supplementary material for: The Impact of Long-Term Clinoptilolite Administration on the Concentration Profile of Metals in Rodent Organisms
Source: Biology (Basel). 2023 Jan 26;12(2):193. doi: 10.3390/biology12020193 (PMC9952783; doi:10.3390/biology12020193)
Supplement: Supplementary file 1 [file biology-12-00193-s001.zip › biology-1819989-supplementary.pdf]

Supplementary material for the paper:

The impact of the long- term clinoptilolite administration on the concentration profile of metals in rodent organisms

Ivan Dolanc [1], Lejla Ferhatović Hamzić [2], Tatjana Orct [3], Vedran Micek [4], Iva Šunić [5], Antonija Jonjić [6], Jasna Jurasović [7], Saša Missoni [8], Miran Čoklo [9] and Sandra Kraljević Pavelić [10]*

[1] Centre for Applied Bioanthropology, Institute for Anthropological Research, 10000 Zagreb, Croatia

[2] Centre for Applied Bioanthropology, Institute for Anthropological Research, 10000 Zagreb, Croatia

[3] Analytical Toxicology and Mineral Metabolism Unit, Institute for Medical Research and Occupational Health, Zagreb, Croatia

[4] Animal Breeding Unit, Institute for Medical Research and Occupational Health, Zagreb, Croatia

[5] Centre for Applied Bioanthropology, Institute for Anthropological Research, 10000 Zagreb, Croatia

[6] Centre for Applied Bioanthropology, Institute for Anthropological Research, 10000 Zagreb, Croatia

[7] Analytical Toxicology and Mineral Metabolism Unit, Institute for Medical Research and Occupational Health, Zagreb, Croatia

[8] Institute for Anthropological Research, 10000 Zagreb, Croatia

[9] Centre for Applied Bioanthropology, Institute for Anthropological Research, 10000 Zagreb, Croatia

[10] University of Rijeka Faculty of Health Studies, Viktora Cara Emina 5, 51000 Rijeka, Croatia

* Correspondence *sandrakp@uniri.hr*; Tel.: (+ 385 51 554 947)

Sadržaj

1. Physiological levels of minerals and metals measured in Wistar rats 3

2. Chemical composition and particle size of tested zeolite materials 3

1. Physiological levels of minerals and metals measured in Wister rats

Supplementary Table S1. Physiological levels of metals and minerals determined from rat plasma and organs as a measure of physiological concentrations of metals and minerals. Concentrations of measured elements in plasma are expressed as mg/l or µg/l, and concentrations of measured elements in organs are expressed as mg/kg or µg/kg.

	Element							
**ORGAN**	**Na**	**Mg**	**Al**	**Si**	**p**	**Ca**	**Fe**	**Mn**
**Concentration**	**mg/l**	**mg/l**	**mg/l**	**mg/l**	**mg/l**	**mg/l**	**mg/l**	**mg/l**
**Plasma**	3274,08	23,86	7,80	3,73	137,77	105,69	8,02	2,55
**Concentration**	**mg/kg**	**mg/kg**	**mg/kg**	**mg/kg**	**mg/kg**	**mg/kg**	**mg/kg**	**mg/kg**
**Brain**	1270,21	199,00	247,00	1,81	3243,51	4602,72	185,89	19,68
**Lung**	1650,21	104,54	637,06	2,84	1974,49	3257,28	120,60	108,12
**Femur**	7681,03	5747,96	952,41	6,18	128332,22	2202,11	313003,38	62,28
**Liver**	622,33	172,96	175,97	2,70	2666,46	4014,76	39,43	450,53
**Ovary**	1076,82	128,08	128,08	4,93	1520,03	2299,95	56,25	70,78
**Kidney**	1689,43	224,34	369,14	1,71	2838,43	2989,42	88,51	99,54

	Element							
**ORGAN**	**Na**	**Mg**	**Al**	**Si**	**p**	**Ca**	**Fe**	**Mn**
**Concentration**	**mg/l**	**mg/l**	**mg/l**	**mg/l**	**mg/l**	**mg/l**	**mg/l**	**mg/l**
**Plasma**	0,80	1660,90	1620,02	42,25	382,24	0,04	0,67	0,22
**Concentration**	**mg/kg**	**mg/kg**	**mg/kg**	**mg/kg**	**mg/kg**	**mg/kg**	**mg/kg**	**mg/kg**
**Brain**	444,85	4,31	2888,26	14112,51	55,27	121,54	0,10	1,60
**Lung**	223,89	7,98	1925,11	26886,11	936,46	319,85	0,87	4,30
**Femur**	570,47	215,72	877,26	215596,06	151,66	108,55	0,74	17,74
**Liver**	2855,95	21,19	5446,44	33970,60	418,85	1429,98	13,20	2,48
**Ovary**	281,48	5,47	1107,54	13412,21	130,35	197,19	0,94	5,25
**Kidney**	977,90	320,88	15719,06	27243,05	154,32	1367,15	28,78	11,95

2. Chemical composition and particle size of tested zeolite materials

2.1. Chemical Composition

SiO_2: 65,0 - 71,3 %
Fe_2O_3: 0,7 - 1,9 %
Al_2O_3: 11,5 - 13,1 %
MgO: 0,6 - 1,2 %
CaO: 2,7 - 5,2 %

Na_2O: 0,2 - 1,3 %
K_2O: 2,2 - 3,4 %
TiO_2: 0,1 - 0,3 %

2.2. Mineralogical Composition

Clinoptilolite 86 %
Quarz 2 %
Cristobalite 6 %
Biotite 5 %
Illite - traces
Feldspar traces
Carbonate-mineral traces

2.3. Ion selectivity

$Cs^+ > NH_4^+ > Pb^{2+} > K^+ > Na^+ > Ca^{2+} > Mg^{2+} > Ba^{2+} > Cu^{2+}, Zn^2$

2.4. Heavy metals

Pb, lead ≤ 14mg / kg
Hg, mercury ≤ 3 mg / kg
As, arsenic ≤ 1.5 mg / kg
Cd, cadmium ≤ 0.5 mg / kg
Co, cobalt ≤ 5 mg / kg
Va, vanadium ≤ 10 mg / kg
Ni, nickel ≤ 20 mg / kg

2.5. Particle size

10% of particles = 1,2 µm
50% of particles = 6,8 µm
90% of particles = 24,2 µm

Supplementary Table S2. Particle diameter size of different zeolites assessed by laser light scattering.

Zeolite	Particle diameter /nm
TMAZ	794,2 ± 29,5
PMA	540,4 ± 15,6
PMA-O2	494,1 ± 11,2
